# Alexithymia Increases Pericranial and Cervical Muscle Tenderness in Women with Migraine

**DOI:** 10.3390/jcm13102772

**Published:** 2024-05-08

**Authors:** Eugenia Rota, Elisa Cavagnetto, Paolo Immovilli, Enrico Frola, Pavel Salari, Nicola Morelli, Alessandro Battaggia

**Affiliations:** 1 Neurology Unit, San Giacomo Hospital, 15067 Novi Ligure, Italy; eugenia.rota.md@gmail.com (E.R.); eli.cavagnetto@gmail.com (E.C.); salaripavel@libero.it (P.S.); 2 Neurology Unit, Guglielmo da Saliceto Hospital, 29121 Piacenza, Italy; 3 IUSTO—Istituto Universitario Salesiano Torino Rebaudengo, 10155 Torino, Italy; enrico.frola@ius.to; 4 Neuroradiology Unit, Guglielmo da Saliceto Hospital, 29121 Piacenza, Italy; nicola.morelli.md@gmail.com; 5 SVEMG—Scuola Veneta di Medicina Generale, 35129 Padova, Italy; a.battaggia@libero.it

**Keywords:** alexithymia, migraine, muscle tenderness, headache, depression, anxiety

## Abstract

**Background/Objectives:** Alexithymia is characterized by a deficit in identifying and communicating feelings. Emerging evidence suggests that alexithymia is highly prevalent in migraine, in a complex interplay with psychiatric comorbidity. Pericranial/cervical muscle tenderness is a remarkable clinical feature in a large proportion of migraine patients. This pilot study aimed at investigating the relationship between alexithymia and pericranial/cervical muscle tenderness in female migraineurs. **Methods:** A total of 42 female patients fulfilling the diagnostic criteria for migraine were enrolled into this pilot, observational, cross-sectional study after informed consent was obtained. Each patient underwent a psychological assessment to identify any alexithymia by means of TAS-20, anxiety/mood comorbidity (by means of STAI-Y1 STAI-Y2, BDI-II), and migraine-related disability (by means of HIT-6), and a physical cranial/cervical musculoskeletal examination. Palpation of pericranial and cervical muscles was carried out in the standardized manner. A Cumulative Muscle Tenderness (CUM) score (0–6) was calculated for each patient. A multivariate analysis was performed to investigate any association amongst the TAS-20 score, the CUM score, and the following covariates: BDI-II, STAI-Y1, STAI-Y2, and HIT-6 scores, age, disease duration, monthly migraine days, and average head pain intensity in the previous three months. **Results:** Overall, 35.6% of the sample had alexithymia. The multivariate analysis detected a linear and independent relationship between the TAS-20 and CUM scores, with a statistically significant (*p* = 0.017) association. **Conclusions:** This pilot study suggests that alexithymia plays a role in increasing pericranial/cervical muscle tenderness in migraine, independently from psychiatric comorbidity. A novel therapeutical approach, targeting alexithymia, may well reduce muscular tenderness in female migraineurs.

## 1. Introduction

Alexithymia (literally, “no words for feelings”) is a term coined by Sifneos in 1973 [1] to describe individuals characterized by a deficit in identifying and communicating feelings and in distinguishing between feelings and bodily sensations [2]. In particular, the inability to adequately recognize physical sensations as the somatic manifestations of emotions can make alexithymic individuals prone to misunderstanding their emotional arousal as signs of disease [2]. Such a multidimensional psychological construct is highly prevalent in chronic pain disorders, where it may affect the clinical phenotype of the pain, entailing deficits in emotion-regulating systems [3].

Emerging evidence suggests that alexithymia is frequent and plays a role in migraine, both episodic and chronic [4,5]. Indeed, it is well known that subjects with migraine often show peculiar personality profiles and psychological traits that may contribute to migraine onset, chronification, and the development of medication overuse [6,7].

Moreover, migraine is frequently comorbid with psychiatric disorders, mainly depression and anxiety, where the relationship is bidirectional [8].

In this context, alexithymia is emerging as a potential characteristic trait of migraine, regardless of disease severity [4]. In chronic migraine, alexithymia seems to be related to medication overuse, traumas, and stressful events [9]. Conversely, other studies report that there is a complex interplay between alexithymia and psychiatric comorbidity in migraine, where alexithymia may correlate with depression and anxiety in migraine sufferers [10,11,12]. Therefore, the question arises as to whether the relationship between alexithymia and migraine is direct or mediated by psychiatric comorbidity.

Neck pain and muscle tenderness are remarkable clinical features in a large proportion of migraine patients [13,14]. There is evidence of statistically significant lower pressure pain threshold values for pericranial muscles in patients with migraine, compared with healthy controls [15]. In a recent study, migraine sufferers showed significant differences, compared with healthy controls, in pressure pain thresholds of some pericranial and cervical muscles and also in cortical excitability and executive functions, highlighting peculiar sensory and pain processing in migraine [16]. A widespread pressure hypersensitivity in the trigeminal cervical area and even in two extra-trigeminal areas of the upper and lower limbs was demonstrated in women with both episodic and chronic migraine [17]. Moreover, an increased number of head/neck myofascial areas able to reproduce this pain were detected in episodic migraine patients, along with an increased cervical pressure hyperalgesia (the latter during the ictal phase), indicating an increased sensitization of the trigeminocervical complex [18,19]. Indeed, growing evidence supports the hypothesis that the tenderness and sensitivity of pericranial and cervical muscles in patients with primary headaches is underpinned by alterations in central pain-processing pathways [20].

Furthermore, pericranial and cervical muscle tenderness (assessed with a cumulative tenderness score—CUM) has been proven to be related to anxiety and depression in various primary headache types [13]. A recent study on migraine patients with neck pain reported that more pronounced cervical musculoskeletal impairments were associated with worse psychological (anxiety/depression) burden [19].

This pilot study aimed at investigating the relationship between alexithymia and muscle tenderness in a sample of female migraineurs. The clinical features with a potential influence on such a relationship were also taken into account, using a statistical analysis with a multivariate regression model, including anxiety and depression as covariates.

## 2. Materials and Methods

This is a pilot, observational, cross-sectional study. All female patients who consecutively referred to the Headache Center of Novi Ligure, Alessandria, Italy, for a first visit within a two-month period and fulfilled the inclusion criteria were enrolled in this study, after written informed consent was obtained. The study was approved by the hospital’s Institution’s Ethics Committee (Protocol Number: Asl20.Neuro.20.01).

Inclusion criteria were as follows: female sex, age between 18–70 years, and a diagnosis of migraine (with or without aura, both chronic and episodic), according to ICHD-III criteria [21].

Exclusion criteria were as follows: impaired ability to provide a detailed history due to a language barrier, an ongoing depressive major episode, relevant medical or surgical comorbidities (a surgical intervention in the previous year), an ongoing migraine attack, the use of any migraine prophylaxis or pain killers for the acute attack in the previous 72 h, and a lack of informed consent.

Forty-two female migraineurs were enrolled, with an average age of 39 ± 16 years (range 18–68 years).

Every patient underwent a semi-structured interview and a psychological assessment. During the interview, demographic data were collected, i.e., age, gender, school years, marital status, occupation, and clinical features, i.e., body mass index, migraine duration, attack frequency, e.g., monthly migraine days, and average pain intensity in the previous three months, measured using a Numerical Rating Scale (NRS, score 0–10).

Then, every patient underwent neuropsychological tests and the Toronto Alexithymia Scale (TAS-20) scale [22] was used to assess alexithymia, the State-Trait Anxiety Inventory (STAI)-Y1 and -Y2 [23] for anxiety, the Beck Depression Inventory (BDI)-II [24] for depression, and the Headache Impact Test (HIT)-6 [25] for migraine-related disability.

Moreover, a physical cranial/cervical musculoskeletal examination was carried out, in the standardized manner, according to the simplified version of a previously described technique [26,27], to determine the presence/absence of muscular tenderness, by the same experienced neurologist (E.R.). The pericranial muscles examined were 1. the masseter, 2. lateral pterygoid, 3. medial pterigoid, and temporal (4. mandibular and 5. cranial insertion). The cervical muscles assessed were the sternocleidomastoid (1. belly and 2. cranial insertion), 3. trapetius, and 4. nuke muscles. Tenderness at palpation was scored in each area from 0 to 3, with 0 indicating normal tone, 1 mild, 2 moderate, and 3 severe tenderness. The scores of pericranial muscles and cervical muscles were added separately and the totals obtained were divided by the number of sites examined. Therefore, a Pericranial Muscle Tenderness Score (PTS) and a Cervical Muscle Tenderness Score (CTS) (range 0–3 each) were calculated for each patient. Lastly, a Cumulative Muscle Tenderness (CUM) score was calculated by the arithmetic sum of the CTS and the PTS (range 0–6).

### Statistical Analysis

A descriptive analysis, based on the mean and standard deviation (S.D.) values and on median and the interquartile range (I.Q.R.), when appropriate, was carried out to determine the demographic and clinical characteristics of the patients. Whenever necessary, the assumption of normality for the distribution of variables was tested using the Shapiro–Wilk and Shapiro–France tests.

A multivariate analysis assessed any association between the CUM score (dependent variable) and the TAS-20 score (primary independent variable), adjusting for the following covariates: demographic (age, marital status, and scholarity in years), metabolic (BMI), and clinical status (disease duration, monthly migraine days, BDI-II, STAI-Y1, STAI-Y2, and HIT-6 scores).

The satisfaction of the normality assumption of both the endpoint values and the residual distribution allowed us to choose an ordinary linear regression (OLS) model.

The strength of association between the covariates and the endpoint was initially studied through a monovariate approach; the selection of clinically relevant variables for patients to be evaluated using multivariate analysis was carried out using inferential criteria with a generous p-cut-off (*p* < 0.20) [28]. The further selection of covariates in the transition from the initial redundant model to the final parsimonious model was performed by adopting the usual confidence levels (statistical significance set at 0.05). The presence of interactions between the variables was investigated so as to study any effect modifiers. Whenever necessary, the variables were modelled in the most suitable way to match the mathematical relationship with the endpoint. We verified that the final model respected the mathematical-statistical assumptions of the OLSs by using the Pregibon test to verify the correct specification; the Breusch–Pagan/Cook–Weisberg test was used to assess the homoskedasticity of the residuals; the identification of influential points was performed via “DFBETA statistic” and their importance was studied by means of sensitivity analysis in models with and without “influential” observations. Lastly, a sensitivity analysis compared the final model to another model enriched with the variables previously excluded during the first selection. All models were compared by Adj-R Squared and AIC (Akaike Information Criterion values).

Data analyses were carried out using the Stata/MP 17.0 6 score, StataCorp LLC 4905 Lakeway Drive College Station TX 7945 USA.

## 3. Results

A total of 18/42 patients (42.8%) had migraine with aura; 35/42 (83.3%) had episodic migraine, and 7 (16.7%) chronic migraine. A total of 22 patients (52.5%) were single, 16 (38%) married or cohabitating, and the remaining 4 (9.5%) were divorced. Table 1 reports the demographic and clinical features, the results of psychological assessments, and the pericranial/cervical tenderness scores.

Overall, 35.6% of the patients had alexithymia: nine patients (21.4%) had a TAS-20 score between 51 and 60 and a diagnosis of a borderline degree of alexithymia, whilst six patients (14.2%), who had a TAS-20 score of >60, had a diagnosis of definite alexithymia.

Table 2A reports the results of the multivariate analysis. Table 2B reports the final results, obtained through the 2B additional model of linear regression with interactions. 

**Figure 1:**
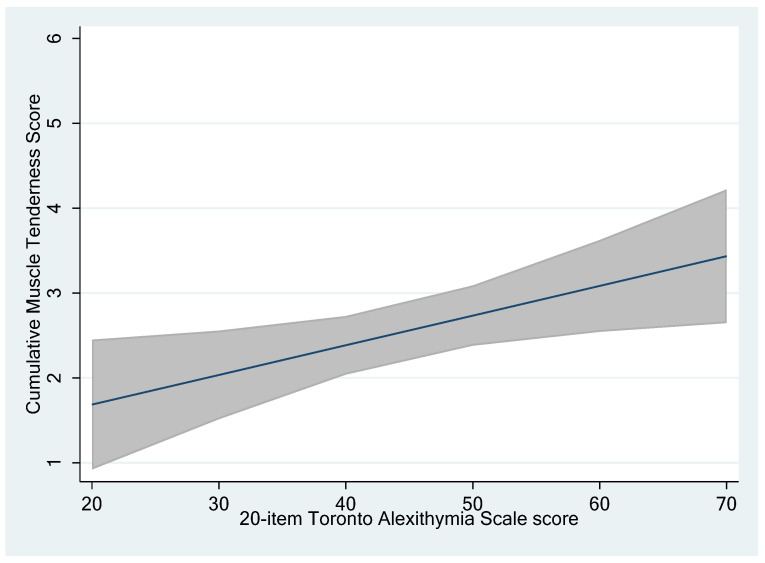
Relationship between the TAS-20 and CUM scores, according to the linear regression model results.

Interestingly, a linear and independent relationship between the TAS-20 and the CUM scores was observed, where each one-point increase in the TAS-20 score predicted a 0.0349-point increase in the CUM score, with a statistically significant (*p* = 0.017) association (Figure 1).

The only one covariate not included in interactions, which significantly affected the CUM score, was the STAI-Y1 score (*p* = 0.035). Indeed, in the final model B with interactions, the STAI-Y1 and CUM scores were linearly statistically significantly associated (*p* = 0.035), where each one-point increase in the STAI-Y1 score predicted a 0.0481-point increase in the CUM score.

**Figure 2:**
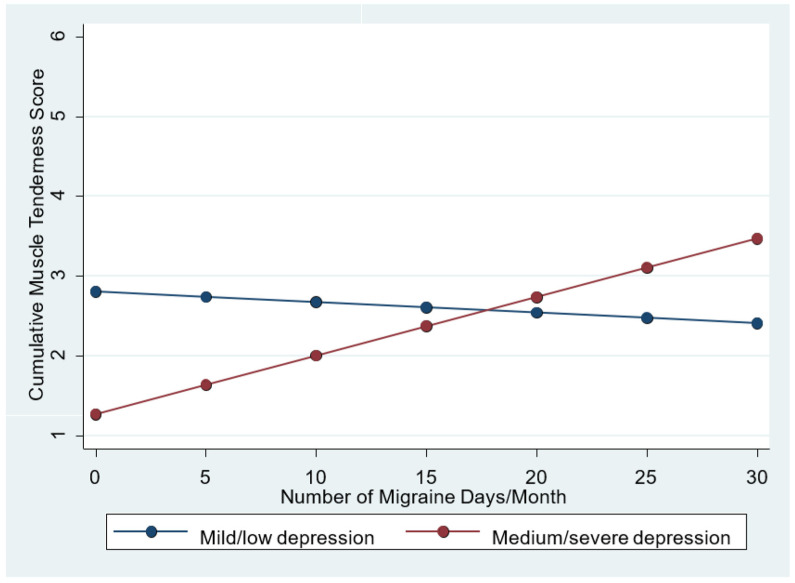
Graphical illustration of the interaction effect between depression and migraine days. Relationship between CUM score and migraine days in the presence of two different levels of depression.

Although the coefficient of the interaction between migraine days or depression (*p* = 0.131) was not statistically significant, an interaction may be suspected based on the following findings:

1. The comparison between model (B) with interaction and model (A) without interaction demonstrates that model B has better information power (AIC model B = 127,781 versus AIC of model A = 130.376).
2. The same comparison reveals that model B has better R-squared values (model R-squared B = 0.3431 compared to model R-squared A = 0.2741).
3. The graphical analysis showed that the residuals of model B have a perfect Gaussian distribution, whilst those of model A seem to be distributed with slight irregularities, although the statistical tests do not suggest a violation of the normality hypothesis in either case. As can be observed in the graph in Figure 2, based on the assumption that such interaction was certain, the effect exerted by the number of monthly migraine days on the CUM score is evident only in patients with moderate–severe depression, whilst in patients with mild or no depression, the number of migraine days does not affect muscle tenderness.

## 4. Discussion

To the best of our knowledge, although preliminarily, this study is the first to suggest that alexithymia plays a significant role in increasing muscle tenderness in migraine, independently from demographic, metabolic, and clinical features and, remarkably, independently from psychiatric comorbidity. Indeed, it seems that alexithymia in female migraineurs has a negative effect on the complex pain phenotype, with a direct and linear relationship with muscle tenderness (*p* = 0.017). Some other studies report that alexithymia may be related to depression and anxiety in migraine sufferers [10,11,12], and that pericranial and cervical muscle tenderness is associated with anxiety and depression [13,19]. Our findings indicate that the psychological feature of alexithymia has a remarkable influence on muscle tenderness in female migraineurs, independently from psychiatric comorbidity. Therefore, this study offers some clarification on this debated issue as to whether the relationship between alexithymia and migraine is direct or underlain by psychiatric comorbidity, supporting the former hypothesis.

Indeed, there is growing evidence in support of alexithymia playing a remarkable role in pain syndromes, mainly in chronic pain [3], and noteworthy is the fact that subjects with migraine often have peculiar personality profiles and psychological traits that may affect the perception of their recurrent pain. Moreover, psychological factors may contribute to migraine onset, chronification, the development of medication overuse, and response to treatment [6,7,29], making it highly plausible that alexithymia influences pericranial and cervical muscle tenderness.

On the other hand, it has been hypothesized that a central sensitization of the trigemino–cervical nucleus underpins neck muscle tenderness and pain in migraineurs [15,18,30].

A biopsychosocial model of migraine has recently been proposed, where genetic predisposition interacts bidirectionally with other biological and environmental factors in determining migraine clinical phenotype and course [29]. A hyperresponsive brain cortex, peripheral and central alterations in pain processing, and comorbidities could play a role, in a complex interplay with psychological factors, including personality traits, psychological features, and traumatic events, as well as with social and lifestyle factors [6,29]. According to the biopsychosocial model, migraine can be considered a dysfunction of sensory processing, characterized by a heightened connection between sensory areas and areas of the limbic system that regulate emotional life and pain processing [29,31].

Interestingly, over time, the construct of alexithymia has been widened to express a more complex emotional dysregulation, involving deficits in the cognitive processing of emotion [32,33]. Structural and functional alterations in brain areas associated with emotional awareness, such as the amygdala, insula, anterior cingulate cortex, fusiform gyrus, and parahippocampal gyrus, were reported in neuroimaging studies for alexithymic individuals [34,35,36].

In this conceptual framework, alexithymia may be hypothesized as the expression of an emotional and cognitive dysregulation in migraine, able to enhance the central sensitization of the trigemino–cervical system, consequently increasing pericranial and cervical muscle tenderness, in a multifaceted interplay where psychological, biological, and environmental factors reciprocally interact.

Indeed, this pilot study evidenced that the STAI-Y1 score also significantly affects the CUM score, i.e., the state of anxiety enhances pericranial/cervical tenderness. This finding is in line with both past [13] and recent evidence [19] of an association between psychiatric comorbidity (for anxiety and depression) and more severe cervical musculoskeletal symptoms, including the tenderness of the pericranial/cervical muscles, in migraine patients.

Considering the interaction between the monthly number of migraine days and depression, which cannot be proven but only suspected by the present study, we may hypothesize that depression acts as an “effect modifier” of the influence that the number of the monthly migraine days has on the CUM score, i.e., only moderate–severe (not mild) depression could be significantly associated with CUM, although it cannot be ruled out that the fact that the coefficient of the interaction does not reach statical significance may be attributable to the small sample size. In fact, such a result has a clinical plausibility, owing to the well-demonstrated association of muscle tenderness both with depression and migraine frequency [13,19]. Moreover, this effect is in agreement with the evidence of the role that depression plays in favoring migraine chronification [37].

However, we are aware that this study does have some limitations. Firstly, it is a pilot study, carried out on a very small, albeit quite homogeneous, sample of female migraine patients.

The observational design (without any randomization) yields weak evidence, supporting the hypothesis of a linear relationship between alexithymia and pericranial/cervical muscle tenderness, which, however, cannot be definitively proven by this study.

Then, the sample numerosity is scarce. Indeed, the fact that some results do not reach statistical significance can only be attributed to the low statistical power or, likewise, to a random error. However, this hypothesis clashes with the clinical plausibility of the findings detected in this sample, which we detailed in the discussion. Secondly, we do not know whether the same results would have been reproduced in a sample of male subjects: this is a limitation concerning the study results’ generalizability.

Moreover, we are aware that the methodology used in this study to assess muscle tenderness in the trigeminal–cervical area has a major shortcoming, in as much as it does not entail an instrumental assessment of the pain pressure threshold, which provides a more objective evaluation of pain sensitivity and has normative values, according to Andersen et al. [15] and other recent studies [16,17,18,19]. However, the pain assessment in this study was carried out with a method that has been widely used and reported in the headache literature since the eighties [13,26,27]. This method was familiar to the investigators [18], easier, and less time-consuming, in clinical practice, than the pain pressure threshold assessment.

Furthermore, since the muscle tenderness examination in our study was performed in the female patients during different phases of the menstrual cycle, without any control, the results may be biased by the influence of the menstrual cycle on pain [38].

Another limitation is that only one instrument was used to measure alexithymia, i.e., TAS-20. Although TAS-20 remains the best standardized and most validated instrument used in past and current research on alexithymia [33], for the assessment of this construct, it would be preferable to adopt a “multi-methodological approach” using multiple tools, such as projective tests or the “observer-rated” Beth Israel Hospital Psychosomatic Questionnaire (BIQ) test [1], or the Toronto Structured Interview for Alexithymia (TSIA) [39].

Despite these drawbacks, this study does have an important strong point, i.e., the inclusion of numerous variables (demographic, metabolic, and clinical) as adjustment covariates, which are potentially capable of exerting, as supported in the literature, direct or indirect effects on CUM as confounding factors.

Lastly, the data that came to light suggest that a novel therapeutical approach which targets alexithymia, such as cognitive behavioral therapy, may improve muscular tenderness in female migraineurs.

## 5. Conclusions

Although further studies are needed to confirm this preliminary finding, the direct and linear relationship between alexithymia and cervical/pericranial muscle tenderness in female patients with migraine clearly indicates a role for alexithymia and the related emotional dysregulation in affecting the pain phenotype in migraine, independently from psychiatric comorbidity.

Therefore, alexithymia may become a target for a novel, non-pharmacological approach, aimed at reducing muscular tenderness in female migraineurs and enhancing their quality of life.

## Figures and Tables

**Table 1:** Demographical, clinical, and neuropsychological features of the sample (*n* = 42).

Variable	Mean	(±S.D.)	Median	IQR
Age	39.32	16.15	39.20	22.35–53.92
Scholarity (years)	14.38	3.41	15.00	13.00–16.25
BMI	22.22	3.65	20.87	19.69–25.00
Monthly migraine days	7.43	7.94	4.50	1.00–20.50
Disease duration (years)	20.76	13.03	17.00	9.00–30.25
NRS score	7.07	1.63	7.00	6.00–8.00
CUM Score	2.54	1.26	2.49	1.52–3.50
TAS-20 Score	44.57	13.37	43.00	34.75–53.50
BDI-II Score	15.86	9.89	15.50	7.00–25.25
STAI-Y1 Score	45.95	10.16	45.00	39.50–52.25
STAI-Y2 Score	51.90	8.89	60.00	51.00–63.00
HIT-6 Score	58.93	7.07	59.00	57.75–63.25

S.D.: Standard Deviation; IQR: Interquartile Range.

**Table 2:** A: Linear regression analysis: final multivariate model without interactions. B *: Linear regression analysis: Additional multivariate model with interactions.

**A**				
**Covariates**	**Monovariate Models**		**Final Multivariate Model**	
	**Coefficient (95% Confidence Intervals)**	***p***	**Coefficient (95% Confidence Intervals)**	***p***
TAS–20 score	0.034 (0.005, 0.062)	0.019	0.034 (0.005, 0.062)	0.023
Body Mass Index (kg/mq)	0.078 (−0.029, 0.185)	0.149	−0.534 (−1.594, 0.525)	0.313
Body Mass Index^2^ (kg/mq)^2^	-	-	0.0146 (−0.008, 0.037)	0.194
Monthly migraine days (number)	0.054 (0.007, 0.102)	0.026	0.0516 (0.0014, 0.102)	0.044
Medium/severe depression (BDI score > 19) vs. Mild/no depression (BDI score ≤ 19)	0.584 (0.210, 1.379)	0.145	−0.940 (−1.937, 0.056)	0.064
Depression#Migraine days (interaction)	-		-	-
STAI-Y1 score	0.045 (0.008, 0.082)	0.018	0.0429 (−0.002, 0.088)	0.06
Age (years)	−0.014 (−0.039, 0.009)	0.231	-	-
Marital status (married/cohabiting vs. single)	0.1939 (−0.655, 1.042)	0.646	-	-
Marital status (divorced vs. single)	1.043 (−0.896, 1.912)	0.469	-	-
Scholarity (years)	−0.0542 (−0.1704, 0.062)	0.352	-	-
Migraine duration (years)	−0.016 (−0.046, 0.015)	0.308	-	-
NRS score	−0.033 (−0.279, 0.213)	0.789		
STAI-Y2 score	0.046 (0.003, 0.089)	0.037	-	-
HIT-6 score	0.069 (0.018, 0.121)	0.01	-	-
_cons	-	-	3.500 (−9.085, 16.085)	0.576
**B**				
**Covariates**	**Monovariate Models**		**Final Multivariate Model**	
	**Coefficient (95% Confidence Intervals** **)**	***p***	**Coefficient (95% Confidence Intervals)**	***p***
TAS–20 score	0.033 (0.005, 0.061)	0.019	0.0349 (0.006, 0.063)	0.017
Body Mass Index (kg/mq)	0.077 (−0.029, 0.185)	0.149	−0.561 (−1.602, 0.479)	0.281
Body Mass Index^2^ (kg/mq)^2^	-	-	0.0151 (−0.006, 0.037)	0.171
Monthly migraine days (number)	0.054 (0.006, 0.101)	0.026	−0.013 (−0.111, 0.085)	0.786
Medium/severe depression (BDI score > 19) vs. Mild/no depression (BDI score ≤ 19)	0.584 (0.210, 1.378)	0.145	−1.534 (−2.785, −0.283)	0.018
Depression#Migraine days (interaction)	-		0.086 (−0.027, 0.200)	0.131
STAI-Y1 score	0.044 (0.008, 0.081)	0.018	0.048 (0.003, 0.092)	0.035
Age (years)	−0.014 (−0.039, 0.009)	0.231	-	-
Marital status (married/cohabiting vs. single)	0.193 (−0.654, 1.042)	0.646	-	-
Marital status (divorced vs. single)	1.042 (−0.896, 1.912)	0.469	-	-
Scholarity (years)	−0.054 (−0.170, 0.062)	0.352	-	-
Migraine duration (years)	−0.015 (−0.045, 0.014)	0.308	-	-
NRS score	−0.032 (−0.278, 0.213)	0.789		
STAI-Y2 score	0.045 (0.002, 0.088)	0.037	-	-
HIT-6 score	0.069 (0.017, 0.121)	0.01	-	-
_cons	-	-	3.816 (−8.545, 16.177)	0.535

* Model 2B presents better values of R-squared (R-squared model B = 0.3431 versus the R-squared model A = 0.2741). Furthermore, from a graphical analysis, the residuals in model B show a perfect Gaussian distribution, while those in model A seem to be distributed with minor irregularities, although in neither case are the statistical tests suggestive of a violation of the assumption of normality.

## Data Availability

Access to all the data in the statistical analysis is available on request.
